# PositiveLinks and the COVID-19 Response: Importance of Low-Barrier Messaging for PLWH in Non-urban Virginia in a Crisis

**DOI:** 10.1007/s10461-021-03294-w

**Published:** 2021-05-11

**Authors:** Breanna R. Campbell, Sabrina Swoger, Alexa Tabackman, Eleanor Hilgart, Benjamin Elliott, Sylvia Coffey, Karen Ingersoll, Rebecca Dillingham, Tabor E. Flickinger

**Affiliations:** 1 Department of Medicine, University of Virginia, Charlottesville, VA USA; 2 School of Medicine, University of Virginia, Charlottesville, VA USA; 3 Mary Baldwin University, Staunton, VA USA; 4 Department of Psychiatry and Neurobehavioral Sciences, University of Virginia, Charlottesville, VA USA; 5 Division of General, Geriatric, Hospital, and Palliative Medicine, School of Medicine, University of Virginia, PO Box 800744, Charlottesville, VA 22908 USA

**Keywords:** HIV/AIDS, COVID-19, Patient-provider communication, Mobile health

## Abstract

PositiveLinks (PL) is an evidence-based mobile health intervention promoting engagement in care for people living with HIV. PL offers secure, in-app patient-provider messaging. We investigated messaging during the early COVID-19 pandemic, comparing messages exchanged between 01/13/2020 and 03/01/2020 (“Pre-COVID”) to messages exchanged between 03/02/2020 and 04/19/2020 (“early COVID”) using Poisson regression. We performed qualitative analysis on a subset of messages exchanged between 02/01/2020 and 03/31/2020. Between “Pre-COVID” and “early COVID” periods, weekly member and provider messaging rates increased significantly. Of the messages analyzed qualitatively, most (53.3%) addressed medical topics, and more than a fifth (21.3%) addressed social issues. COVID-related messages often focused on care coordination and risk information; half of COVID messages contained rapport-building. PL patients (“members”) and providers used in-app secure messaging to reach out to one another, identifying needs, organizing receipt of healthcare resources, and strengthening patient-care team relationships. These findings underscore the importance of low-barrier messaging during a crisis.

## Resumen

PositiveLinks (PL) es una intervención de salud móvil basada en evidencia que promueve la participación en la atención de las personas que viven con el VIH. PL ofrece mensajería segura entre paciente y proveedor dentro de la aplicación. Investigamos la mensajería durante la fase temprana de la pandemia de COVID-19, comparando los mensajes intercambiados entre el 13 de enero y el 1 de marzo del 2020 ("pre-COVID") con los mensajes intercambiados entre el 2 de marzo y el 19 de abril del 2020 ("COVID") usando el modelo de regresión de Poisson. Realizamos un análisis cualitativo de un subconjunto de los mensajes intercambiados entre el 1 de febrero y el 31 de marzo del 2020. Entre los períodos "pre-COVID" y "COVID," los índices semanales de mensajes de los miembros y proveedores aumentaron significativamente. De los mensajes analizados cualitativamente, la mayoría (53.3%) abordó temas médicos y más de una quinta parte (21.3%) abordó temas sociales. Los mensajes relacionados con COVID a menudo se centraron en la coordinación de la atención y la información sobre riesgos; la mitad de los mensajes sobre COVID presentó contenido relacionado con el establecimiento de buenas relaciones. Los pacientes de PL ("miembros") y los proveedores utilizaron la mensajería segura dentro de la aplicación para comunicarse entre sí, identificar necesidades, organizar la recepción de recursos de atención médica, y fortalecer las relaciones entre el equipo de atención y el paciente. Este estudio subraya la importancia de facilitar un fácil acceso a la mensajería durante una crisis.

## Introduction

The first known case of COVID-19 (coronavirus disease 2019, henceforth “COVID”) in the Commonwealth of Virginia was announced on March 7, 2020 [1]. When the World Health Organization declared COVID a pandemic on March 11, Virginia Governor Ralph Northam declared a State of Emergency for the Commonwealth [1]. The first case of COVID in Charlottesville was reported on March 16, and non-urgent ambulatory visits at the University of Virginia (UVA) Health System were cancelled in favor of telemedicine services [2]. Issued on March 23, Executive Order 53 closed certain non-essential businesses, limited gathering size, and closed K-12 schools; on March 30, Executive Order 55 instituted a state-wide Stay-At-Home Order in effect until June 10, 2020 [3, 4]. During this time, the patient population at the UVA Ryan White Clinic experienced significant life changes.

People living with HIV (PLWH) need consistent medical care. Medication adherence and engagement in care are associated with improved clinical outcomes [5]. PLWH face many challenges that can put them at risk for gaps in care, including socioeconomic barriers. PositiveLinks (PL) is a mobile health (mHealth) intervention that provides a platform to deliver evidence-based practices for linkage to and engagement in care to PLWH in Virginia [5]. PL has been offered as usual care at the University of Virginia Ryan White clinic since 2017. Our PL coordinator and staff continuously monitor PL use by its “members,” tailoring the intervention through ongoing quality improvement efforts to better meet patients' needs.

The PL platform includes daily queries related to medication adherence, mood, and stress to encourage self-monitoring; a community message board for secure, anonymous communication with other clinic-connected PLWH; and a secure method for hosting telemedicine appointments with members. PL further provides a unique form of secure messaging, a feature highly favored by all PL users—especially those at greatest risk for disengagement from care [6]. PL members can exchange secure messages through the app with their providers and PL administrators. PL providers include physicians, nurse practitioners, social workers, case managers, psychologists, pharmacists, and others, reflecting our team approach to care delivery. The PL administrators are primarily responsible for app and phone functioning concerns; they do not participate in medical care delivery.

Secure messaging between patients and their healthcare providers is increasingly prevalent and can improve the management of chronic medical conditions [7]. Our prior work indicates that PL reaches a vulnerable population with a high prevalence of low literacy, low socioeconomic status, and racial/ethnic minority status [8]. These patient populations also tend to have low uptake of traditional electronic medical record systems’ patient portals and are at greater risk for adverse health events related to the COVID-19 pandemic [9, 10].

With the arrival of novel coronavirus to the United States, many medical appointments were canceled or delayed; COVID disrupted in-person health care. Telemedicine appointments were initiated and rapidly increased to bring care to patients in their homes [2, 11]. During this time, the PL members at the UVA Ryan White Clinic continued using the PL app, messaging their providers and administrators. While technology offered opportunities to maintain connections to care in a time of disruption, it was unknown how messaging would be used in unprecedented circumstances. The objectives of this quality improvement project were (1) to evaluate the rates of secure messaging by members, providers, and administrators and (2) to investigate the content and function of the messages exchanged through the PL platform in the early COVID-19 pandemic.

## Methods

This mixed-methods analysis utilized quantitative methods to compare messaging rates between “pre-COVID” and “early COVID” time periods, then employed qualitative methods to investigate differences in messaging content between patients (“members”) and their Ryan White care team across the “pre-COVID” and “early COVID” time periods. This evaluation of a program offered as usual care at the clinic using de-identified data was determined by our Institutional Review Board (IRB) to be a quality improvement project and therefore not subject to full committee review.

### Messaging Rates

We compared messages exchanged between 01/13/2020 and 03/01/2020 (“Pre-COVID”) to messages exchanged between 03/02/2020 and 04/19/2020 (“early COVID”) to observe potential differences in messaging patterns. Enrollment in the PositiveLinks program is ongoing, and the number of active members changes dynamically over time. Members can be “deactivated” from PL when members elect to leave the clinic or the PL program. Members are not routinely “deactivated” without their initiative unless they stop interacting with the app for 12 weeks at a time despite PL staff outreach. Consequently, messaging data is reported by week (Monday through Sunday) and averaged by member counts for each week. Therefore, we determined the cut-off of 03/02/2020 by considering both the onset of COVID activity in Virginia (March) and the messaging data's weekly division.

The two quantitative datasets encompass seven weeks. We tabulated weekly sums of total messages sent, total active members enrolled in PL, total active providers, and total active administrators, comparing the total number of messages exchanged per group in the “Pre-COVID” and “early COVID” periods. In order to account for changes in number of active members per week as described above, we constructed messaging rates by diving the total number of weekly messages sent by each role (member, provider, and administrator) by the total number of individuals in these roles. Member posting rates to the community message board were also determined. We calculated minimum, median, mean with standard deviation, and maximum messaging and posting rates per week. These values were averaged over each 7-week period to create two overall datasets. We analyzed our non-normal data by Poisson regression in R Studio running R version 4.0.2.

### Messaging Content

Utilizing Dedoose (Dedoose Version 8.0.35, web application for managing, analyzing, and presenting qualitative and mixed method research data (2018); Los Angeles, CA: SocioCulturalResearchConsultants, LLC www.dedoose.com), we analyzed a subset of the messages to determine what topics were discussed by members, administrators, and providers during the observed time frames. We adapted a codebook from a prior study of PL messaging [12] to include additional codes capturing COVID-related themes, coding messages from 02/01/2020 to 02/29/2020 (the qualitative "Pre-COVID" dataset) and 03/01/2020 to 03/31/2020 (the qualitative "early COVID" dataset). These qualitative datasets were 4-week subsets within the 7-week time periods of the quantitative analysis. A non-coding researcher (RD) anonymized data by replacing message sender and recipient identities with randomly assigned numbers and by removing potentially identifying information from the message text, thus protecting privacy before analysis.

**Fig. 1:**
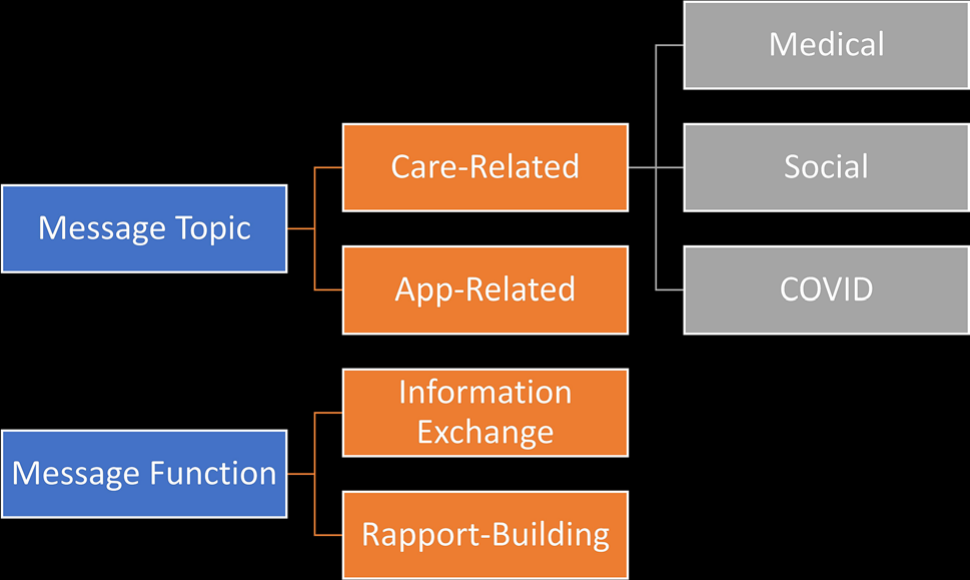
Hierarchy of qualitative codes

While most messages were in English, we included Spanish-language messages where present. Figure 1 displays the hierarchy of qualitative codes. Topic categories were app-related or care-related, as developed in our prior work [12]. App-related messages included those about managing the PL app or membership, technical difficulties, phone payment coordination, setting up meetings with PL administrators for phone or app support, and feedback about the app. Care-related messages were either medical, social, or specifically concerning COVID. Medical messages included clinic-based patient care, medications, appointments, member outreach, and physical and mental health information. Social messages addressed social determinants of patient health, including insurance, transportation, housing, food, utilities, disability, finances, and work. COVID messages were those with any mention of COVID, coronavirus, or other references to the pandemic. These could overlap with either medical (e.g., symptoms) or social (e.g., loss of a job, work modification to avoid exposure) codes.

Message function was also analyzed, determining if messages merely exchanged information or went further to build rapport. Information exchange messages were utilitarian in nature, whereas rapport-building messages included psychosocial components that expressed emotions and sought to build relationships. Each message received at least one topic code and at least one function code, but could receive more than one if relevant (i.e., topic and function codes were not mutually exclusive). The codebook was applied to all messages within the qualitative dataset so that frequencies could be determined. Five coders performed the coding independently (BC, EH, SS, AT, TF). During codebook development, the coding team held weekly meetings to discuss any adaptations needed, reach agreement on the new COVID-related codes, and resolve any coding discrepancies or ambiguities by consensus, under the supervision of the senior coder (TF). Graphical representations of qualitative data were created using R.

## Results

### Messaging Rates

There were 472 PositiveLinks members at the beginning of the “Pre-COVID” dataset. During the time periods evaluated, provider and administrator counts remained steady, but the overall number of members rose as new members were enrolled each week. By the end of the timeframe, the total member count rose to 497; sixteen of these additional members enrolled in PL during the “early COVID” period. No members were "deactivated" during the "early COVID" period.

During the “Pre-COVID” period, 4560 total messages were exchanged; 5702 total messages were exchanged during the “early COVID” period (a 25% increase). Members sent 1817 messages in the 7-week "Pre-COVID" period and 2413 messages in the 7-week "early COVID" time frame. “Pre-COVID,” 83.9% of member-sent messages were delivered to providers, increasing to 87.5% in the “early COVID” time frame. Providers and administrators also demonstrated an increase in messaging, whether sending or receiving messages; virtually all provider-sent and administrator-sent messages were delivered to patients. Comparatively, member community message board posts decreased from 1722 member posts “Pre-COVID” to 1658 posts in the “early COVID” period (a 4% drop).

When evaluating by weekly messaging rates per group, the mean weekly member-sent message rate increased from 0.55 to 0.68 (β = 0.211, p = 0.01). Overall member community message board posting dropped minimally, but this finding was nonsignificant. The weekly provider-sent message rate increased from 4.32 to 5.91 (β = 0.314, p = 0.0005) while the increase in weekly administrator-sent message rate was non-significant (β = 0.002, p = 0.98).

### Messaging Content

We analyzed 6668 messages qualitatively: 30% of total messages were app-related, 53.3% addressed medical topics, and 21.3% addressed social topics. Regarding message function, 86.9% of total messages contained information exchange, and 41.0% contained rapport-building. Table 1 shows code frequencies by sender type with examples of typical messages, chosen from the entire period. For all quotes, we have retained the message senders' original spelling, grammar, and punctuation. The majority of member-sent and provider-sent messages were on medical topics, while administrator-sent messages were usually app-related. Members most often sent messages to providers (n = 2372); there was minimal exchange of messages between providers and administrators (n = 5). For all sender types, most messages contained information exchange. Providers had the highest proportion of rapport messages [n = 1051 (46%), with one message to an administrator], followed by members [n = 1213 (42%), with 967 sent to providers], and then administrators [n = 473 (32%), with one message sent to a provider].

**Table 1:** Message topic and function frequencies by sender type across the entire qualitative dataset

Message code	Member sender (n = 2902)	Provider sender (n = 2278)	Administrator sender (n = 1488)	Example
Topic				
App-related	504 (17%)	65 (3%)	1429 (96%)	Member to Administrator: “I got a moto g7 I need to transfer my phone but I’m not going to do it until I speak to you”
Medical	1877 (62%)	1716 (75%)	47 (3%)	Member to Provider: “I got 1 more pill at this point. Never run out b4 I come to clinic, so I was wondering if that particular script was put in..”
Social	723 (25%)	651 (29%)	47 (3%)	Member to Provider: “I’m having transportation issues today and I’m having to put my car in the shop… I will have to reschedule my appointment with you today”
Function				
Information exchange	2342 (81%)	2029 (89%)	1423 (96%)	Member to Administrator: “I don’t see where I can set it to use fingerprint again. I uninstalled the app and reinstalled it and still don’t see it. Help”
Rapport	1213 (42%)	1051 (46%)	473 (32%)	Provider to Member: “how are you? I’ve been so excited to see that your viral loads have been undetectable—congratulations!!!”

**Fig. 2:**
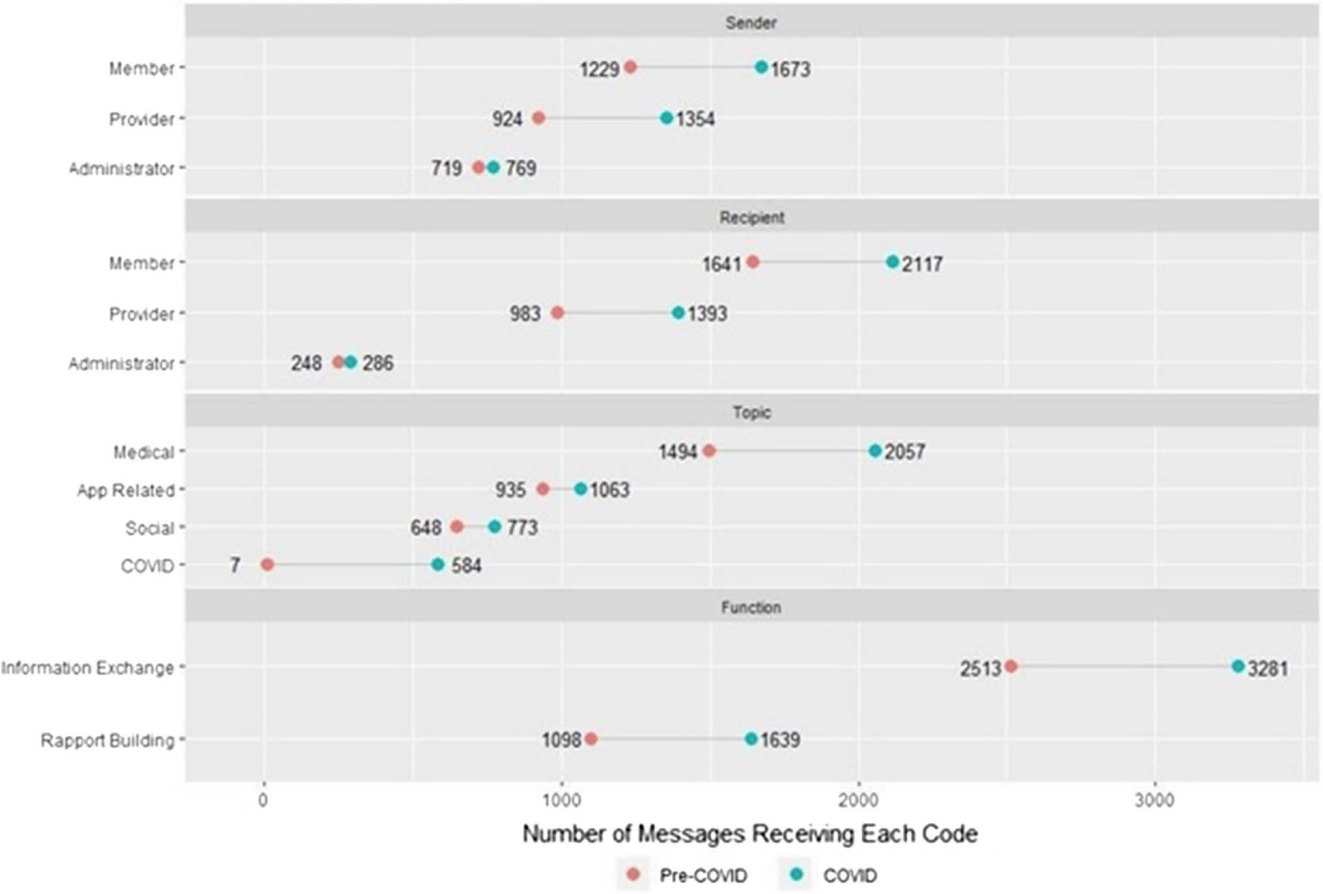
Messaging trends, pre-COVID versus early COVID

Figure 2 demonstrates overall code frequencies, distinguished between "Pre-COVID" and "early COVID" datasets. During the “Pre-COVID” timeframe, 2872 total messages were exchanged; 3796 messages were exchanged during the “early COVID” timeframe. We further subcategorized the COVID-related messages; Table 2 shows the topic, function, and subtypes of the COVID-related messages with examples. There were seven COVID-related messages sent during the “Pre-COVID” timeframe, which were primarily risk/precaution messages, anticipating the spread of COVID to Virginia. All other COVID-related messages were sent in the designated “early COVID” timeframe. The most frequent subtype of the COVID-related messages was care coordination, followed by risk/precautions and social impact. Half of the COVID-related messages contained rapport-building.

**Table 2:** All COVID-related messages (n = 591; 7 from the pre-COVID dataset and 584 from the COVID dataset) with code frequencies and examples from messages sent by members

Message code	Count (%)	Example
Topic		
App-related	11 (64%)	Member to Administrator: “Sorry I’ve been missing few days. It’s been crazy trying to keep up with everything that us going on and trying to manage things at home. My mind has been out of focus and forgot to log in few days to track and answer the questions”
Medical	344 (58%)	Member to Provider: “I wanted to see if we could have an appointment via telephone in the next few days. I understand that you are not doing in person appointments.”
Social	151 (26%)	Member to Provider: “I am a little worried because I can’t work because of my health issues. So, my income (extra) has stopped. Very concerned about the house purchase now.”
Function		
Information exchange	487 (82%)	Member to Provider: “I keep hearing I am at more risk but that doesn’t make sense to me since I do not have cd4 counts below 500 ever. Am I right or am I missing something?”
Rapport building	297 (50%)	Member to Provider: “Hi, I just want to let you know I’m very sorry for being so rude and just getting up and leaving. I had worked all night, and was tired. I keep getting bad acid reflex, and its very painful. Anyways I'm truley sorry and thanks for seeing me. You'r the best”.
COVID subtypes		
Care coordination	255 (43%)	Member to Provider: “Hello, I was wondering if we could set up a FaceTime (zoom, google chat, etc) appointment? Let me know. I hope you’r well. Hopefully will see you before I go!”
Risks/precautions	130 (22%)	Member to Provider: “quick question about covid 19 and my hiv diagnosis. Am I still considered immune depressed given my very normal and consistent CD4 counts?”
Social impact	130 (22%)	Member to Provider: “I’m fine on food right now that’s not my concern. I’m still behind on rent because I’ve been so sick and now with this covid 19 mess my job is closed until who knows when. So my biggest concern was housing assistance.”
Concern for others/well wishes	101 (17%)	Member to Provider: “Thank you so much, I really do appreciate this. It seems [other provider name] is doing pretty well so far though this crazy crisis! Bless your heart for doing what you do, you are an inspiration! Please be super careful and stay awesome!”
Emotional/mental health impact	91 (15%)	Member to Provider: “Great!!!! To be honest I'm a little beside myself. Worried about my children, wife and self….but….we will get thru this!!!<br>Take care of yourself and family!!!!”
Physical symptoms	27 (5%)	Member to Provider: “I had all the symptoms 5 weeks later my cough is almost gone and I almost have my voice back!! It’s crazy!!! I am about 90 percent better almost there!!”

**Fig. 3:**
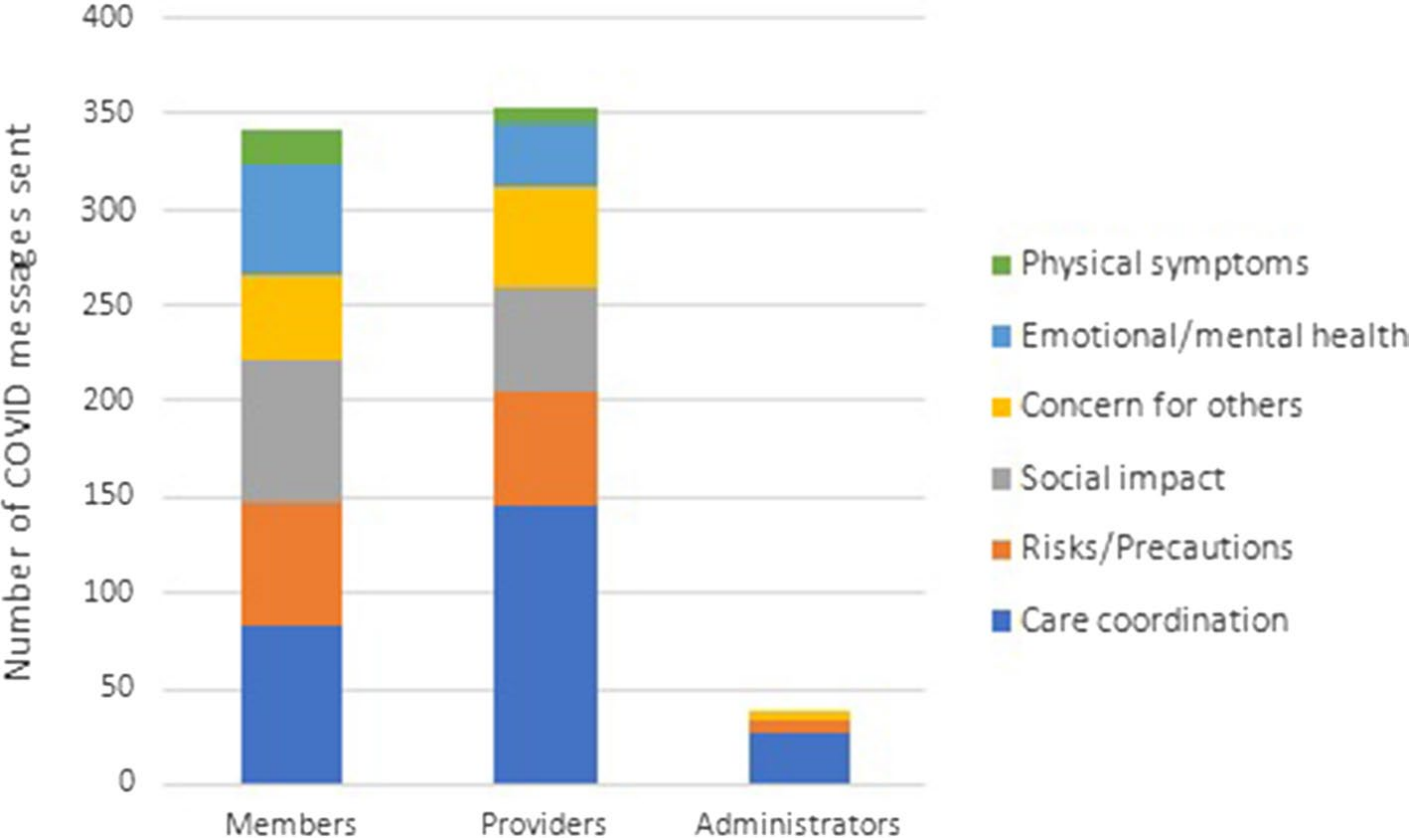
COVID topic subtypes by sender

We evaluated code co-occurrences to characterize the COVID messages further. Figure 3 shows the number of messages sent for each COVID subtype by sender roles. Care coordination was the most frequent message subtype regardless of role, with providers sending most of the care coordination messages (n = 145 out of total 255). After care coordination, members most frequently sent messages discussing the social impact of COVID (n = 75), whereas providers most often discussed COVID risks and precautions (n = 60). PL administrators also sent messages regarding COVID risks/precautions (n = 6) and communicating concern for others/well wishes (n = 5). As noted in Table 2, physical symptoms were least often discussed, but members sent messages regarding physical symptoms twice as frequently as did providers (n = 18 and n = 9, respectively).

Providers often reached out to members regarding appointments while expressing concern: “Hi [name]—given the reschedule of our appt tomorrow, I wanted to check in to see how you’re doing? Would you like to do a phone check in tomorrow morning? Hope you’re staying well, [provider name]”. However, providers also sent messages solely for outreach (“I just wanted to reach out and let you know that our clinic is here to support during this time”) or encouragement (“We care for you, I know this is so hard. You are being so brave in the midst of such a hard time”). Members both responded to and initiated conversations. Member-initiated conversations most often arranged appointments, requested medication refills, and informed care team members of needs relating to social determinants of health (see Table 2 for sample messages).

Across the time periods, we observed additional important topics among non-app-related, member-initiated messages. One member contacted her psychologist regarding an acute decompensation in mental health, which enabled the care team to provide just-in-time response. On one occasion, a member notified their physician of substance use relapse and sought to arrange treatment. Another member contacted her psychologist to aid in HIV status disclosure to her significant other (the patient’s later messages revealed that this conversation went “better than well. He was compassionate, loving and caring. He made love him even more”). Not all messages were positive. In one instance, a member asked a provider to aid in reassignment from one clinician to another, expressing concern with provider availability and responsiveness: “Anyone can make a mistake and overlook something but this has become ridiculous!” A different member utilized messaging to apologize for in-clinic behavior, stating in part, “Im truely sorry and thanks for seeing me. Your the best.” Of additional interest, another member reached out to their non-medical case manager requesting patient-centered information regarding U = U (“undetectable = untransmittable,” referencing that PLWH with a serum viral load < 200 copies/mL cannot transmit HIV through sexual interactions). The member sought “a link or pdf or something you could send me with a clear, concise statement of facts, info, science and/or evidence about u = u and modern HIV treatment that I can share to help clarify and dispel their ignorance.”

Overall, 194 messages were in Spanish, with 47% sent by members, 45% sent by providers, and 7% sent by PL staff. Most messages were medical (89%), followed by social (13%) and app-related (4%). Only 4 Spanish-language messages were COVID-related, 3 of which dealt with care coordination, and 1 expressed concern for other/well wishes. Tables 1 and 2 include the Spanish messages among the total numbers of messages.

## Discussion

Utilizing both quantitative and qualitative methods, we evaluated secure messaging in our mHealth app, PositiveLinks, before and after the arrival of COVID-19 in Virginia, finding marked increases in messaging overall and COVID-specific care coordination. This quality improvement project underscored the importance of low-barrier messaging to enable PLWH in a time of crisis.

Between "Pre-COVID" and "early COVID" periods in 2020, we observed a significant increase in member-sent messaging rates, specifically identifying that more messages were delivered to providers (care team members) as opposed to administrators who support phone service and app functioning. By contrast, we observed an overall decrease in member posting on the community message board. Our findings suggest that connection specifically with the care team was of increasing importance to our patients, especially after we entered the COVID-19 pandemic. We further identified significantly increased utilization of secure messaging by PL providers to provide outreach to PLWH, showing that secure in-app messaging enables providers to connect with patients. Having found increased messaging rates among members and providers, we then examined the content of a subset of exchanged messages to understand better what role in-app messaging served during this critical period. The PL messaging feature was used to address medical and social topics as well as app-related issues. For comparison, our prior analysis of PL messages sent November 2017–January 2018 were categorized as 57.6% app-related, 34.3% medical, and 12.4% social [12]. By contrast, our qualitative 2020 dataset contained more medical (53.3%) and social (21.3%) messages and fewer app-related messages (30.0%). Improvements in technology literacy (including mobile device and mobile app use) as well as PL app improvements may have contributed to the observed trend. For COVID-related messages, the most frequent subtype among all users was care coordination. These messages helped members navigate disruptions in their appointments, set up telemedicine consultations, maintain access to their medications, and remain connected with their providers. Our secure, low-barrier messaging permitted the clinical team to maintain connections with patients through the uncertainty and difficulties posed by the arrival of COVID-19 to Virginia.

Messaging was also used to address the social impact of COVID-19, such as problems with work, housing, or food access. By communicating these issues to the Ryan White care team, our patients were able to receive various resources addressing social determinants of health, including food/grocery cards, utilities assistance, and housing support [13] The secure, low barrier messaging enabled our team to quickly address developing issues, lessening COVID-induced and COVID-exacerbated barriers to care.

PL messaging served a role in communicating key public health information regarding the COVID-19 pandemic. Almost a quarter (22%) of COVID-related messages were related to precautionary measures, including hand-washing, mask wearing, and social distancing. This occurred during a time in which precautionary recommendations were frequently being revised, and the public may have been receiving conflicting information from various sources. Low-barrier messaging with health providers during the initial outbreak of COVID-19 provided patients with reliable, updated knowledge on key public health preventative measures to slow viral spread.

We also noted members reporting rising emotional and mental health needs through the COVID period. These needs were addressed through PositiveLinks messaging either directly by mental health clinicians who are PL providers or through coordination of local mental health resources for members. Here again, the secure, low barrier messaging provided an additional avenue for delivering valuable healthcare resources to our patients during the pandemic, further promoting engagement with care and lessening the effect of barriers to in-person care.

Investigating messaging function enabled us to identify if messaging was merely utilitarian or served a more personal purpose. We observed that most messages exchanged information, but half also featured rapport building. Of note, the rapport was often reciprocal, with patients not only receiving rapport from providers but also expressing concern for providers' wellbeing and appreciation for the care team. For comparison, our prior analysis of PL messages sent November 2017–January 2018 were categorized as information exchange (87.3%) and rapport-building (33.8%) [12]. Overall, there was more rapport-building (41%) in 2020 and, notably, the COVID-related messages demonstrated more rapport-building (50%) than seen in the earlier 2020 dataset. Rapport may be particularly important when in-person relationships are disrupted, and electronic communication becomes the primary means through which patient-provider connections can be maintained. In times of uncertainty and distress, rapport building can address patient needs for emotional connection [14]. In addition to patient distress, the COVID-19 pandemic has contributed to worsening burnout and adverse mental health for clinicians [15, 16]. Improved quality of in-person patient-clinician communication has been associated with reduced clinician burnout [17]. If this extends to electronic communication, rapport building through messaging may be of benefit both to patients and their care team and presents an area for future study.

Finally, we observed that conversations across the studied period were often initiated by PL members themselves, whether to organize appointments, to seek information regarding COVID-19, to communicate needs, or to build rapport. Occasionally, messages expressed negative sentiments or apologies to the care team; in other instances, patients took charge to direct their care goals. Electronic health records (EHRs) may provide an option for patients to take initiative to interact with care team members. However, in a review of studies examining uptake and use of EHRs by PLWH, low uptake of EHRs was noted due to poor technology access, lower technological literacy, and privacy concerns [18]. By contrast, the secure, low-barrier messaging feature in PL was regularly employed by patients to seek and participate more fully in care, forging or repairing relationships with their care team. Patients elected to utilize in-app PL messaging for self-advocacy and empowerment in the time of COVID-19, similar to observations in our prior work [6, 12].

The findings of this quality improvement project were used in the clinic to optimize the PL messaging function during the early COVID-19 pandemic to meet the needs of patients and the care team. The uptake of messaging for care coordination facilitated the scheduling and logistics of telehealth appointments to maintain continuity of care. We started sharing secure links for telehealth appointments through PL messages and adjusted integration so that audio and video could be turned on automatically, thus reducing technology barriers. The process for eligibility documentation was also refined so that the messaging could be used to keep members informed about which documents they needed and a direct contact person to assist them. A new cohort notification feature was added to allow messages from the clinic to be transmitted to all PL users, which would display on their app home page instead of their inbox, so that important information could be shared effectively, such as COVID-related announcements, updates, or clinic events.

### Limitations

Messages were analyzed chronologically without threading between sender and recipient, making it occasionally difficult to follow the flow of conversations. We do not have clinical information for patients using the PL messaging, such as incidence of COVID-19 infections. We cannot say whether the risk/precaution information given to patients averted exposures, nor can we comment on other possible impacts on care. It is also unknown whether patterns seen in PL messaging are generalizable to other tools, such as MyChart or other patient portals. Long term follow-up would be needed to determine whether COVID-related disruption of in-person care has adverse effects on viral suppression and whether use of PL messaging mitigated these effects. Follow-up analysis at a later time point could also address changes in COVID-related messaging topics over time. Our focus was on the early phase of the pandemic, but communication patterns have likely changed over time as the pandemic itself continues to change. For example, new topics may emerge related to vaccination. Examination of messaging topics and frequencies at subsequent time points would be an area of interest for future analysis.

## Conclusion

PositiveLinks, developed with our patients and continually iterated over time in response to patient input, is adapted to low literacy and intentionally designed to be accessible to patients, including those with lower economic and educational attainment and/or members of minority racial/ethnic groups [5]. For these patients, barriers to communication with their health care team may not be alleviated by commercially available health system portals [9, 18]. PL patients and providers used in-app secure messaging to reach out to one another. They identified needs, organized receipt of healthcare resources, and perhaps strengthened patient-care team relationships. Patients took initiative to direct their care goals, in part utilizing messaging for self-advocacy and empowerment. Overall, there is a growing body of literature which underscores the importance of low-barrier messaging during a crisis, as seen in veteran and young adult populations with mental health concerns [19, 20], and our work contributes to this literature for PLWH.
